# Mining for novel antibiotics in the age of antimicrobial resistance

**DOI:** 10.1111/1751-7915.13662

**Published:** 2020-09-02

**Authors:** Zulema Udaondo, Miguel A. Matilla

**Affiliations:** ^1^ Department of Biomedical Informatics University of Arkansas for Medical Sciences Little Rock AR 72205 USA; ^2^ Department of Environmental Protection Estación Experimental del Zaidín Consejo Superior de Investigaciones Científicas Prof. Albareda 1 Granada 18008 Spain

## Abstract

The misuse of antimicrobials is causing an alarming increase in the appearance of antibiotic‐resistant microorganisms. In this context, the identification of novel antibiotics against new targets and with low rates of resistance development is a major global challenge. In this article, we highlight a number of recent articles that exploit a variety of *in vitro*, *in vivo* and *in silico* state‐of‐the‐art approaches to identify and develop new antimicrobials. Rapid progress in this research field will be crucial to combating a global health problem, antimicrobial resistance, that is expected to be the leading cause of death by 2050.

## Graphical abstract

The emergence of antibiotic‐resistant microorganisms urges the development of novel antimicrobials that are effective against new targets and with low rates of resistance appearance. The combined use of modern multidisciplinary approaches including synthetic biology, genomics, metagenomics, proteomics, transcriptomics, metabolomics and computational tools are enabling progress in this re‐emerging research field.

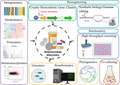

The COVID‐19 pandemic has brought back into focus the global vulnerability of our society to infectious diseases (Brüssow, 2020). Among them, antimicrobial resistance (AMR) is currently one of the major global health concerns, with current predictions indicating that it will cause 10 million deaths annually by 2050 and a global cost of 100 trillion USD (Trotter *et al*., 2019). The emergence of AMR correlates with the misuse of antibiotics, and its rise is not only restricted to animal and human pathogens but also affects economically relevant phytopathogens (Davies and Davies, 2010; Robbins *et al*., 2017; Sundin and Wang, 2018). The mechanisms of AMR are generally associated with (i) the acquisition of resistance genes through horizontal gene transfer, (ii) the mutation of cellular targets, (iii) the production of alternative antimicrobial‐insensitive targets, and (iv) the enzymatic inactivation of the antimicrobials (Davies and Davies, 2010; Robbins *et al*., 2017; Sundin and Wang, 2018). Consequently, there is an urgent need to develop new antibiotics that not only present low rates of resistance development but also have novel mechanisms of action to avoid cross‐resistance with currently used drugs. In fact, more than 70% of the global projects in the preclinical antibacterial pipeline focus on searching for antibiotics with new targets (Theuretzbacher *et al*., 2020).

Actinobacteria continue to be the main source of clinically used antimicrobials (van Bergeijk *et al*., 2020), but genome mining strategies are revealing the enormous genetic potential of alternative microbial taxa to discover novel antibiotics (Blin *et al*., 2019; Sharrar *et al*., 2020). This fact is exemplified in a recent study in *Microbial Biotechnology* where the combination of genome mining and phylogenetics of core polyketide synthase domains led to the discovery of an uncharacterized 35 kb biosynthetic gene cluster (BGC) in a fungal endophyte isolated from a traditional Chinese medicine plant. Importantly, subsequent high‐resolution mass spectrometry and NMR spectroscopy analyses linked this BGC to the production of a novel anti‐*Candida* antifungal polyketide, lijiquinone (Cain *et al*., 2020). Unfortunately, the majority of the BGCs involved in natural product synthesis remain silent under standard laboratory culture conditions and there is a lack of knowledge on the regulatory mechanisms and environmental cues that activate cryptic BGCs (van Bergeijk *et al*., 2020). As a result, in the current genomics‐based antimicrobial discovery context, a variety of experimental approaches are employed to awake the expression of cryptic BGCs. For example, recent advances have been focussed on microbial co‐cultivation, high‐throughput elicitor screenings, synthetic biology strategies, the development of new chassis microorganisms for heterologous expression or paired metatranscriptomics and metabolomics of native microbial communities (Rutledge and Challis, 2015; Matilla *et al*., 2018; Zhang *et al*., 2019; van Bergeijk *et al*., 2020). In this regard, a recent report published in *Microbial Biotechnology* used a novel bacteriophage recombinase system, Redαβ7029, to efficiently exchange the natural promoters of the seven cryptic polyketide synthase/non‐ribosomal peptide synthetase BGCs present in the genome of the antimicrobial producer and plant‐growth‐promoting bacterium *Paraburkholderia megapolitana* DSM 23488 (Zheng *et al*., 2020). This elegant genome editing strategy resulted in the successful activation of two silent BGCs, BGC5 and BGC9. The subsequent combination of high‐resolution mass spectrometry and NMR spectroscopy allowed the determination of the chemical structure of the products of BGC9, which were named haereomegapolitanin A and B. These molecules belong to a novel class of lipopeptides and, based on the domain organization of the core biosynthetic proteins encoded in BGC9, the authors proposed a model for the biosynthesis of both haereomegapolitanins. Although the identified haereomegapolitanins did not show antimicrobial properties against the tested bacteria and fungi, the real impact of the study lies in the enormous potential of the Redαβ7029‐based expression strategy for the efficient manipulation of Burkholderiales strains (Zheng *et al*., 2020). Burkholderiales are emerging as rich sources of natural products (Blin *et al*., 2019). However, the majority of the BGCs present in their genomes seem to be cryptic (Zheng *et al*., 2020) and the lack of efficient genetic tools for the manipulation of native Burkholderiales species is currently hindering the identification of bioactive natural products with potential medical and agricultural importance.

On another note, high‐throughput screenings (HTSs) of chemical libraries have been shown to represent efficient approaches for the identification of synergistic combinations of antimicrobials (i.e. the combined efficacy of two antimicrobials is greater than the sum of their individual effects; Wambaugh *et al*., 2020). Remarkably, given the low rates in the discovery of new antimicrobials nowadays, the synergistic combination therapy not only offers the possibility to revitalize the use of existing antibiotics, but also increases the activity spectrum of the treatment, minimizes host toxicity and reduces the occurrence of AMR as synergistic drugs generally act against different cellular targets (Tyers and Wright, 2019). Indeed, synergistic therapies have been shown to be effective to combat AMR bacteria (Song *et al*., 2020a). In this context, an article in *Microbial Biotechnology* revealed that the quinones hypocrellin A, B and C strongly inhibit mycelium formation, biofilm formation and virulence of different *Candida* species and *C. albicans* strains (Song *et al*., 2020b). Importantly, the authors showed that hypocrellins have a synergistic effect with the antifungal nystatin in an AMR *C. albicans* isolate. In addition, synergistic effects of hypocrellins with additional antifungal compounds (e.g. fluconazole, amphotericin B and voriconazole) were also shown to decrease the minimal inhibitory concentration (MIC) values of these antimicrobials by up to 16‐fold. The authors hypothesize that the observed synergistic effect may be a consequence of the effect of hypocrellins on biofilm formation as well as during *C. albicans* morphological transition, key steps during the infection process of *C. albicans* (Song *et al*., 2020b).

High‐throughput screenings have been also successfully used for the discovery of antimicrobials with novel mechanisms of action (Kitamura *et al*., 2018). In this respect, the screening of a library of 33 000 small molecules resulted in the identification of SCH‐79797 as a potent antibacterial agent against important Gram‐negative and Gram‐positive pathogens including *Acinetobacter baumannii*, *Staphylococcus aureus*, *Neisseria gonorrhoeae* or *Enterococcus faecalis* with undetectable AMR development (Martin *et al*., 2020). Significantly, SCH‐79797 effectiveness was demonstrated against several AMR isolates, while the inability to isolate SCH‐79797‐resistant mutants hindered the elucidation of the mechanism of action (MoA) of this antibacterial. To overcome this difficulty, in a recently published *Cell* article the authors combined quantitative imaging, thermal proteome profiling, CRISPR interference sensitivity, metabolomics, enzymology and quantitative flow cytometry to reveal that SCH‐79797 targets both membrane integrity and folate metabolism (Martin *et al*., 2020). Subsequent assays demonstrated that this molecule, due to its double antibacterial activities, outperforms the effects of certain different antibiotic combinations in the treatment of AMR bacteria. The chemical basis of the unique dual MoA of SCH‐79797 was elucidated, aspect that was key for the development of chemical derivatives with enhanced antibacterial activities. One of these derivatives, Irresistin‐16 (IRS‐16), exhibited enhanced antibacterial activity in comparison with SCH‐79797, and it was efficient for treating *N. gonorrhoeae* in a mouse vaginal infection model with low host toxicity. The authors highlighted that combining several MoA in a single chemical scaffold may be an efficient approach to treat infections caused by AMR pathogens (Martin *et al*., 2020).

Despite AMR being a rising threat, recent efforts aimed at discovering and developing new antimicrobials in public and private sectors have not advanced significantly, with only 12 new antibiotics (or combinations) being approved in the last two decades (Kaufmann *et al*., 2018). However, the rapid progress in the development of integrative platforms based on the combination of genomics, metagenomics, metabolomics and bioinformatics is facilitating the analysis of thousands of microorganisms in order to mine the antimicrobial biosynthetic potential of specific taxonomic groups and ecosystems (Sharrar *et al*., 2020; van der Hooft *et al*., 2020). In addition, recent algorithmic advancements in modelling neural networks have started to influence the paradigm of drug discovery (Stokes *et al*., 2020). Thus, deep learning approaches on chemical libraries demonstrated that the combination of *in silico* predictions and empirical methodologies can lead to the discovery of antibiotics with novel scaffolds effective against clinically relevant bacterial pathogens, including AMR bacteria and persister cells (Stokes *et al*., 2020). Altogether, future research on antimicrobial discovery will benefit from (meta)genomics and metabolomics as well as from modern machine learning approaches. The rapid progress in these methodologies will potentially increase the success rate of antimicrobial discovery and will reduce the total cost of antibiotic development, which is currently estimated at 200 million USD per bioactive molecule (Ribeiro da Cunha *et al*., 2019).

## Conflict of interest

The authors declare no conflict of interest.
